# Bariatric Surgery and Incident Development of Obesity-Related Comorbidities

**DOI:** 10.1001/jamanetworkopen.2025.30787

**Published:** 2025-09-09

**Authors:** Amanda L. Bader, Jesse Y. Hsu, Maria S. Altieri, Charles M. Vollmer, James D. Lewis, David E. Kaplan, Nadim Mahmud

**Affiliations:** 1 Department of Surgery, University of Pennsylvania Health System, Philadelphia; 2 Department of Biostatistics, Epidemiology and Informatics, University of Pennsylvania, Philadelphia; 3 Perelman School of Medicine, University of Pennsylvania, Philadelphia; 4 Division of Gastroenterology, University of Pennsylvania Health System, Philadelphia

## Abstract

**Importance:**

As obesity rates rise in the US, managing associated metabolic comorbidities presents a growing burden to the health care system. While bariatric surgery has shown promise in mitigating established metabolic conditions, no large studies have quantified the risk of developing major obesity-related comorbidities after bariatric surgery.

**Objective:**

To identify common metabolic phenotypes for patients eligible for bariatric surgery and to estimate crude and adjusted incidence rates of additional metabolic comorbidities associated with bariatric surgery compared with weight management program (WMP) alone.

**Design, Setting, and Participants:**

This retrospective, multicenter cohort study used data from the Veterans Health Administration (VHA) Corporate Data Warehouse, which incorporates data from 128 VHA centers. Participants were adults 18 years or older with a body mass index (BMI; calculated as weight in kilograms divided by height in meters squared) of 30 or higher and at least 1 of 5 metabolic comorbidities or with a BMI of 35 or higher who underwent bariatric surgery (Roux-en-Y gastric bypass or sleeve gastrectomy) or enrolled in the WMP (called MOVE!) between January 1, 2008, and December 31, 2023. Patients with all 5 comorbidities at baseline or missing key data were excluded.

**Exposure:**

Bariatric surgery vs WMP.

**Main Outcome and Measures:**

Incident diagnosis of any of 5 metabolic comorbidities: type 2 diabetes (T2D), hypertension, hyperlipidemia, obstructive sleep apnea (OSA), and metabolic dysfunction–associated steatotic liver disease (MASLD).

**Results:**

Among 269 470 veterans, 263 657 were enrolled in the WMP and 5813 underwent bariatric surgery. Patients included 232 196 males (87.1%) and had a median (IQR) age of 57 (47-64) years. Median (IQR) follow-up time was 112.9 (79.5-145.4) months. At 5 years, incidence rates per 1000 person-years were 8.89 for hypertension, 9.67 for hyperlipidemia, 4.29 for T2D, 3.99 for OSA, and 2.44 for MASLD in the WMP group. For the bariatric surgery group, incidence rates per 1000 person-years were 3.35 for hypertension, 4.85 for hyperlipidemia, 1.06 for T2D, 3.43 for OSA, and 2.01 for MASLD. Bariatric surgery was associated with a statistically significant lower risk of incident T2D (79.2% lower; hazard ratio [HR], 0.21 [95% CI, 0.18-0.26]), hypertension (58.8% lower; HR, 0.41 [95% CI, 0.33-0.51]), hyperlipidemia (50.5% lower; HR, 0.49 [95% CI, 0.42-0.58]), OSA (56.9% lower; HR, 0.43 [95% CI, 0.35-0.52]), and MASLD (40.4% lower; HR, 0.60 [95% CI, 0.49-0.73]) compared with the WMP. Results were consistent in a subgroup analysis of only female veterans.

**Conclusions and Relevance:**

In this cohort study, bariatric surgery was associated with a significantly lower risk of developing major metabolic comorbidities compared with the medical WMP. This finding supports the relevance of bariatric surgery as a durable approach for obesity-related risk mitigation.

## Key Points

**Question:**

In patients with obesity, does bariatric surgery reduce the risk of developing metabolic comorbidities compared with a medical weight management program?

**Findings:**

In this cohort study of 269 470 veterans, bariatric surgery was associated with a significantly lower risk of incident type 2 diabetes, hypertension, hyperlipidemia, obstructive sleep apnea, and metabolic dysfunction–associated steatotic liver disease, compared with the weight management program, over 5 years.

**Meaning:**

Bariatric surgery may be a durable option for lowering the risk of developing metabolic comorbidities in patients with obesity.

## Teaser

This cohort study evaluates the incidence rates of type 2 diabetes, hypertension, hyperlipidemia, obstructive sleep apnea, and metabolic dysfunction–associated steatotic liver disease among US veterans who underwent bariatric surgery and were enrolled in the Veterans Health Administration medical weight loss program.

## Introduction

Obesity poses a major threat to public health, with more than one-third of the US adult population classified having obesity or severe obesity.^1,2^ It is associated with 18% of deaths in the US, likely mediated through the development of metabolic comorbidities, including cardiovascular disease, diabetes, hypertension, obstructive sleep apnea (OSA), and certain cancers.^3,4^ Bariatric surgery procedures, including sleeve gastrectomy (SG) and Roux-en-y gastric bypass (RYGB), are indicated in patients with a body mass index (BMI; calculated as weight in kilograms divided by height in meters squared) of 30 or higher with at least 1 major metabolic comorbidity.^5^ These surgeries result in sustainable weight loss with an acceptable safety profile and are associated with improvement in and even remission of key metabolic comorbidities—most notably, hypertension, diabetes, hyperlipidemia, and metabolic dysfunction–associated steatotic liver disease (MASLD).^2,6,7^ Despite these benefits, only 0.5% to 1.0% of eligible patients receive bariatric surgery.^8^

There are several reasons for underutilization of bariatric surgery, including lack of education among patients and clinicians about safety and efficacy, limited access due to socioeconomic factors, and lack of insurance coverage.^9,10^ Additionally, the US Food and Drug Administration has approved several medications for weight management in the past 5 years; increasing demand for antiobesity medications may further decrease demand for bariatric surgery.^11^ However, a potentially modifiable contributor to bariatric surgery underutilization may be the inability to anticipate the development of key outcomes tailored to patients who present with different comorbidity profiles. Limited data quantify comorbidity diversity among those eligible for bariatric surgery, and there are no large studies of incident metabolic comorbidities after surgery. While preoperative discussions often focus on weight loss or remission benefits, the risks of forgoing surgery have not been framed in terms of additional comorbidities. Better understanding of these issues may lead to more comprehensive counseling and potentially greater bariatric surgery utilization.

To address these knowledge gaps, we aimed to (1) identify common metabolic phenotypes for patients eligible for bariatric surgery and (2) estimate crude and adjusted incidence rates of additional metabolic comorbidities associated with bariatric surgery vs the alternative of a weight management program (WMP) alone.

## Methods

### Design and Data Cohort Creation

This retrospective cohort study used the established dataset of the Veterans Health Administration (VHA) Corporate Data Warehouse (CDW), which incorporates data from 128 VHA centers.^12,13^ The Corporal Michael J. Crescenz Philadelphia Veterans Affairs Medical Center Institutional Review Board approved this study and waived the informed consent requirement because deidentified data were used. We followed the Strengthening the Reporting of Observational Studies in Epidemiology (STROBE) reporting guideline.

We included patients aged 18 years or older, with a BMI of 35 or higher or 30 or higher, and with at least 1 of 5 major metabolic comorbidities (hypertension, hyperlipidemia, type 2 diabetes [T2D], OSA, or MASLD) who were referred to the VHA’s medical WMP—called MOVE!—or who received bariatric surgery (RYGB or SG) between January 1, 2008, and December 31, 2023. The BMI and comorbidity criteria are in accordance with the 2022 American Society for Metabolic and Bariatric Surgery eligibility criteria for bariatric surgery.^14^ The WMP encourages veterans to increase physical activity and transition to healthy eating habits. The program combines behavioral-based dietary and physical activity self-management through individual and group counseling as well as telephone care. Additional treatments, such as weight loss medication, intensive outpatient care, and residential treatment, may be offered as adjuncts to the core program.^15^ Once a veteran is referred to the WMP, a baseline assessment is performed followed by several weeks of educational group sessions with individualized follow-up.

The study index date was either the bariatric surgery date or the risk-set matched date after program referral. Patients were excluded if they had all 5 metabolic comorbidities at baseline or were missing key data at baseline, including BMI and laboratory data such as lipid panel or hemoglobin A_1c_ (HbA_1c_) (eFigure 1 in Supplement 1). The latter criterion was used to minimize misclassification of exposures and outcomes, consistent with prior studies.

### Primary Exposure and Covariates

The primary exposure was weight loss intervention: bariatric surgery vs WMP. Bariatric surgery procedures were identified using *Current Procedural Terminology* codes, summarized in eTable 1 in Supplement 1, including those from VHA Fee Basis tables to capture surgeries performed outside of the VHA.^12,16,17,18^ The CDW consults table was queried to identify all referrals to the WMP.

Detailed patient data were also collected, including demographics (age, sex, and self-identified race and ethnicity), BMI, Alcohol Use Disorders Identification Test–Concise (AUDIT-C) score, laboratory data (low-density lipoprotein [LDL] cholesterol and HbA_1c_), and diagnosis of a major metabolic comorbidity (hypertension, hyperlipidemia, T2D, OSA, or MASLD). The AUDIT-C (score range: 0-12, with a score of ≥5 indicating a positive screen for unhealthy alcohol use) is a 3-item alcohol screening tool that reliably identifies individuals with hazardous drinking or active alcohol use disorders; this tool is administered to US Department of Veterans Affairs (VA) patients at least once a year.^19^ Race and ethnicity (reported as Asian, Black, Hispanic, Hispanic Black, Native Hawaiian or Other Pacific Islander, White, and other [including American Indian or Alaska Native]) were included in this study because of a known association between race and all of the outcomes of interest.

For patients enrolled in the WMP prior to receiving bariatric surgery, time in the program was calculated from the referral date to surgery date. Baseline variables were considered the most recent data available prior to the index date, up to a maximum of 2 years. Data on BMI, LDL cholesterol, HbA_1c_, and AUDIT-C score were obtained in a time-updated fashion throughout the follow-up period and were updated at a maximum frequency of every 6 months; for windows without updated data, a last-value-carried-forward approach was used. Major metabolic comorbidities were defined using validated algorithms, based on *International Classification of Diseases, Ninth Revision* and *International Statistical Classification of Diseases and Related Health Problems, Tenth Revision* codes, and laboratory data (eTable 2 in Supplement 1).^20,21,22,23,24,25^

### Outcomes

The primary outcome was the incident diagnosis of any of the 5 major metabolic comorbidities (hypertension, hyperlipidemia, T2D, OSA, or MASLD) in patients without these conditions at baseline. Ascertainment of these comorbidities using validated algorithms is detailed in eTable 2 in Supplement 1.^20,21,22,23,24,25^

### Statistical Analysis

Descriptive statistics were computed as medians and IQRs for continuous data and as frequencies and percentages for categorical data in the unmatched cohort. Statistical comparisons of bariatric surgery and WMP groups were made using Wilcoxon rank-sum and χ^2^ tests, respectively. Crude incidence rates for incident metabolic comorbidities were calculated in the unmatched cohort for each of the 5 metabolic comorbidities. Patients with the comorbidity of interest at baseline were excluded before incidence was calculated per 1000 person-years, stratified by receipt of bariatric surgery or not.

Given the goal of estimating the risk of different metabolic comorbidities associated with the WMP vs bariatric surgery, we created a series of 5 patient subcohorts who did not have a given comorbidity at baseline. To identify similar patients at baseline other than receipt of bariatric surgery vs WMP, we used a risk-set propensity score matching (PSM) approach. PSM entailed matching the bariatric surgery group to the WMP group using time-varying data that also corresponded to equivalent times spent in the WMP. The WMP controls only included patients who remained in the program and never had surgery. For instance, a patient who underwent bariatric surgery and spent 6 months in the WMP prior to surgery would be propensity score–matched to a patient enrolled in the WMP who had also spent 6 months in the program at the time of matching (eFigure 2 in Supplement 1). Propensity scores were calculated using the following covariates, which were selected a priori, in logistic regression models: age, race and ethnicity, sex, BMI, LDL cholesterol, HbA_1c_, AUDIT-C score, and comorbidity burden. To account for comorbidity burden, each model from propensity score–matched cohorts included the remaining 4 metabolic comorbidities (not used as the outcome) as time-updated covariates. A 3:1 nearest-neighbor matching approach was used with caliper widths set to 0.05 SDs of the logit of the propensity score. Covariate balance was assessed before and after PSM by computing standardized mean differences (SMDs), with an absolute SMD less than 0.1 reflecting adequate balance.^26^ These data were presented for each of the 5 subcohorts.

Next, to estimate the association of bariatric surgery with incident comorbidity development over a 5-year time horizon, time-to-event analysis was performed with data right-censored at death or maximum follow-up. First, unadjusted Kaplan-Meier curves were plotted in the prematching cohort, and log-rank tests were used for statistical comparison of curves. Additionally, Cox proportional hazards regression model was used to estimate the cause-specific hazard of a given incident metabolic comorbidity in both prematched (crude) and matched (adjusted) cohorts. For example, in the subcohort of patients without diabetes at baseline, the relative hazard of incident diabetes was estimated. A shared frailty term was used in the Cox proportional hazards regression models to account for matching, which thus accounted for time in the WMP for each matched set.^27^ Hazard ratios (HRs) and 95% CIs were reported for each model, and the cumulative incidence of each metabolic comorbidity was visualized from crude and adjusted models using heat plots. Cause-specific Cox proportional hazards regression models were used to estimate relative hazards, given their interpretability for assessing covariate effects. Subdistribution hazard models from Fine and Gray analysis were used only to estimate cumulative incidence. Because the focus was on relative risks rather than absolute risks, subdistribution hazard models were used only to estimate cumulative incidence. The proportional hazards assumption was evaluated using Schoenfeld residuals; no violations were observed in the adjusted Cox proportional hazards regression models.

For descriptive statistics, when comparing patients in the bariatric surgery group to patients in the WMP group, a α = .05 threshold for statistical significance was used, and all tests were 2-sided. For outcomes where each of the 5 metabolic comorbidities were studied, a Bonferroni-adjusted α = .01 threshold was used to account for multiple comparisons. All statistical analyses were performed using Stata/BE 18.0 (StataCorp LLC). Data were extracted from the CDW using structured query language (SQL), and data cleaning and analysis were performed using SQL and Stata.

Given that the VHA cohort is predominantly male yet most patients who undergo bariatric surgery in the general population are female, a risk-set PSM was used for a subgroup analysis. This analysis was isolated to females to provide specific risk estimates for this subgroup.

## Results

### Cohort Characteristics

Overall, 269 470 patients were included in the study, of whom 232 196 (87.1%) were males and 34 496 (12.9%) were females, with a median (IQR) age of 57 (47-64) years. Among these patients, 1352 (0.5%) identified as Asian, 52 809 (20.1%) as Black, 37 502 (14.3%) as Hispanic, 10 246 (3.9%) as Hispanic Black, 2749 (1.0%) as Native Hawaiian or Other Pacific Islander, 140 169 (53.3%) as White, and 2959 (1.1%) as other race and ethnicity. Table 1 details cohort characteristics prior to PSM. A total of 263 657 were enrolled in the WMP and never underwent surgery, while 5813 patients underwent bariatric surgery (1803 [0.7%] received RYGB; 4010 [1.5%] received SG), of whom 3417 were previously enrolled in the WMP.

**Table 1.** Cohort Characteristics Before Propensity Score Matching

Characteristic	Patients, No. (%)			*P* value
	Total (N = 269 470)	WMP group (n = 263 657)	Bariatric surgery group (n = 5813)	
Surgery type				
RYGB	1803 (0.7)	NA	1803 (31.0)	NA
Sleeve	4010 (1.5)	NA	4010 (69.0)	NA
Follow-up time, median (IQR), mo	112.9 (79.5-145.4)	113.2 (80.0-145.5)	96.9 (58.1-135.6)	<.001
Time in the WMP, median (IQR), mo	36.6 (16.4-69.8)	36.6 (16.4-69.8)	NA	NA
Race and ethnicity^a^				
Asian	1352 (0.5)	1331 (0.5)	21 (0.4)	<.001
Black	52 809 (20.1)	51 797 (20.1)	1012 (18.8)	<.001
Hispanic	37 502 (14.3)	36 737 (14.3)	765 (14.2)	<.001
Hispanic Black	10 246 (3.9)	10 051 (3.9)	195 (3.6)	<.001
Native Hawaiian or Other Pacific Islander	2749 (1.0)	2696 (1.0)	53 (1.0)	<.001
White	140 169 (53.3)	137 255 (53.3)	2.914 (54.1)	<.001
Unknown	15 297 (5.8)	14 906 (5.8)	391 (7.3)	<.001
Other^b^	2959 (1.1)	3124 (1.1)	39 (0.7)	<.001
Sex				
Male	232 196 (87.1)	228 560 (87.5)	3636 (66.7)	<.001
Female	34 496 (12.9)	32 682 (12.5)	1814 (33.3)	<.001
Age, median (IQR), y	57 (47-64)	57 (47-64)	49 (41-57)	<.001
Smoking status				
Current	84 986 (32.4)	83 721 (32.6)	1265 (23.8)	<.001
Former	73 907 (28.2)	72 476 (28.2)	1331 (25.0)	<.001
Never	103 335 (39.4)	100 615 (39.2)	2719 (51.2)	<.001
Baseline BMI, median (IQR)	36.8 (34.0-40.6)	36.7 (33.9-40.4)	44.2 (40.0-49.4)	<.001
Baseline hypertension	162 323 (60.9)	158 560 (60.7)	3763 (69.0)	<.001
Baseline hyperlipidemia	149 626 (56.1)	146 173 (56.0)	3453 (63.4)	<.001
Baseline T2D	82 022 (30.8)	83 692 (32.0)	2046 (37.5)	<.001
Baseline MASLD	86 272 (32.3)	83 692 (32.0)	2580 (47.3)	<.001
Baseline OSA	61 158 (22.9)	57 914 (22.2)	3244 (59.5)	<.001
Baseline AUDIT-C score, median (IQR)^c^	0 (0-1)	0 (0-1)	0 (0-1)	.001
Baseline HbA_1c_, median (IQR), %	6.1 (5.6-7.0)	6.1 (5.6-7.1)	5.9 (5.5-6.5)	<.001
Baseline LDL cholesterol, median (IQR), mg/dL	104.0 (81-130)	104.0 (81-130)	102.0 (79-126.8)	<.001

Abbreviations: AUDIT-C, Alcohol Use Disorders Identification Test–Concise; BMI, body mass index (calculated as weight in kilograms divided by height in meters squared); HbA_1c_, hemoglobin A_1c_; LDL, low-density lipoprotein; MASLD, metabolic dysfunction–associated steatotic liver disease; NA, not applicable; OSA, obstructive sleep apnea; RYGB, Roux-en-Y gastric bypass; T2D, type 2 diabetes; WMP, weight management program.

SI conversion factor: To convert HbA_1c_ to proportion of total hemoglobin, multiply by 0.01; and LDL cholesterol to millimoles per liter, multiply by 0.0259.

^a^ Self-identified by patients and obtained from Veterans Health Administration Corporate Data Warehouse.

^b^ Includes American Indian or Alaska Native.

^c^ AUDIT-C score range: 0 to 12, with a score of 5 or greater indicating a positive screen for unhealthy alcohol use.

Compared with the WMP group, the bariatric surgery group was younger (median [IQR] age, 57 [47-64] vs 49 [41-57] years), had higher baseline median (IQR) BMI (36.7 [33.9-40.4] vs 44.2 [40.0-49.4]), and higher baseline prevalence of all 5 comorbidities (eg, T2D: 83 692 [32.0%] vs 2046 [37.5%]). Baseline median (IQR) HbA_1c_ was lower (5.9% [5.5%-6.5%] vs 6.1% [5.6%-7.1%] [to convert to proportion of total hemoglobin, multiply by 0.01]) in the bariatric surgery vs WMP group, and baseline median (IQR) LDL cholesterol was also lower (102.0 [79-126.8] mg/dL vs 104.0 [81-130] mg/dL [to convert to millimoles per liter, multiply by 0.0259]). Among the entire cohort, the most common combinations of the 5 baseline metabolic comorbidities were none (39 612 [14.7%]); hypertension and hyperlipidemia (27 000 [10.0%]); and hypertension, hyperlipidemia, and T2D (26 813 [10.0%]).

After risk-set PSM for each subcohort, excellent covariate balance was achieved for all variables, with reduction in SMD to within 0.05 (eFigure 3 in Supplement 1). Of the patients who underwent surgery and were eligible for matching, 3971 (68.3%) were matched and 1842 (31.7%) were unmatched. However, among the unmatched patients, 3964 (68.2%) had 3 or more metabolic comorbidities at baseline and thus had limited opportunity to be matched.

### Association Between Bariatric Surgery and Incident Comorbidity Development

**Figure 1.**
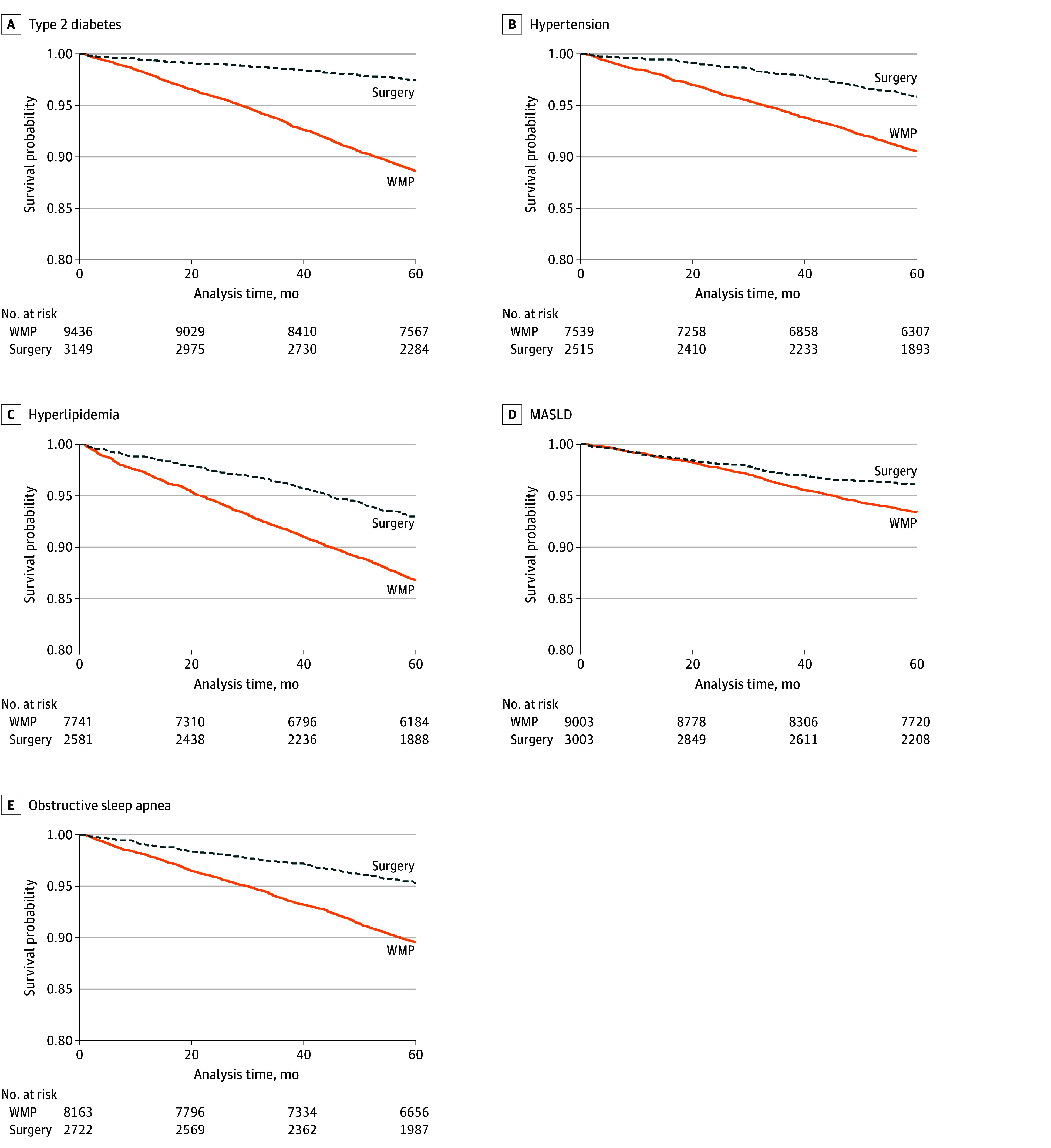
Kaplan-Meier Curve for Each Outcome MASLD indicates metabolic dysfunction–associated steatotic liver disease; WMP, weight management program.

Median (IQR) follow-up time was 112.9 (79.5-145.4) months overall, with 113.2 (80.0-145.5) months in the WMP group and 96.9 (58.1-135.6) months in the bariatric surgery group. Median (IQR) time in the WMP for patients who eventually underwent surgery was 36.6 (16.4-69.8) months. There were significant differences in survival distributions (each log-rank *P* < .001), with bariatric surgery demonstrating a lower risk of metabolic comorbidities over time (Figure 1).

Crude, unadjusted cumulative incidence rates of each comorbidity in the the WMP and bariatric surgery groups are shown in eFigure 4 in Supplement 1. At 5 years, the incidence rates per 1000 person-years were 8.89 for hypertension, 9.67 for hyperlipidemia, 4.29 for T2D, 3.99 for OSA, and 2.44 for MASLD in the WMP group. In the bariatric surgery group, the incidence rates per 1000 person-years were 3.35 for hypertension, 4.85 for hyperlipidemia, 1.06 for T2D, 3.43 for OSA, and 2.01 for MASLD. Unadjusted HRs are presented in Table 2.

**Table 2.** Unadjusted and Adjusted Hazard Ratios for Each Outcome

Comorbidity	Unadjusted HR (95% CI)	*P* value	Adjusted HR (95% CI)	*P* value
**T2D**				
Bariatric surgery vs WMP	0.25 (0.22-0.29)	<.001	0.21 (0.18-0.26)	<.001
**Hypertension**				
Bariatric surgery vs WMP	0.38 (0.35-0.45)	<.001	0.41 (0.33-0.51)	<.001
**Hyperlipidemia**				
Bariatric surgery vs WMP	0.51 (0.47-0.56)	<.001	0.49 (0.42-0.58)	<.001
**OSA**				
Bariatric surgery vs WMP	0.85 (0.77-0.95)	.003	0.43 (0.35-0.52)	<.001
**MASLD**				
Bariatric surgery vs WMP	0.81 (0.73-0.92)	.001	0.60 (0.49-0.73)	<.001

Abbreviations: HR, hazard ratio; MASLD, metabolic dysfunction–associated steatotic liver disease; OSA, obstructive sleep apnea; T2D, type 2 diabetes; WMP, weight management program.

**Figure 2.**
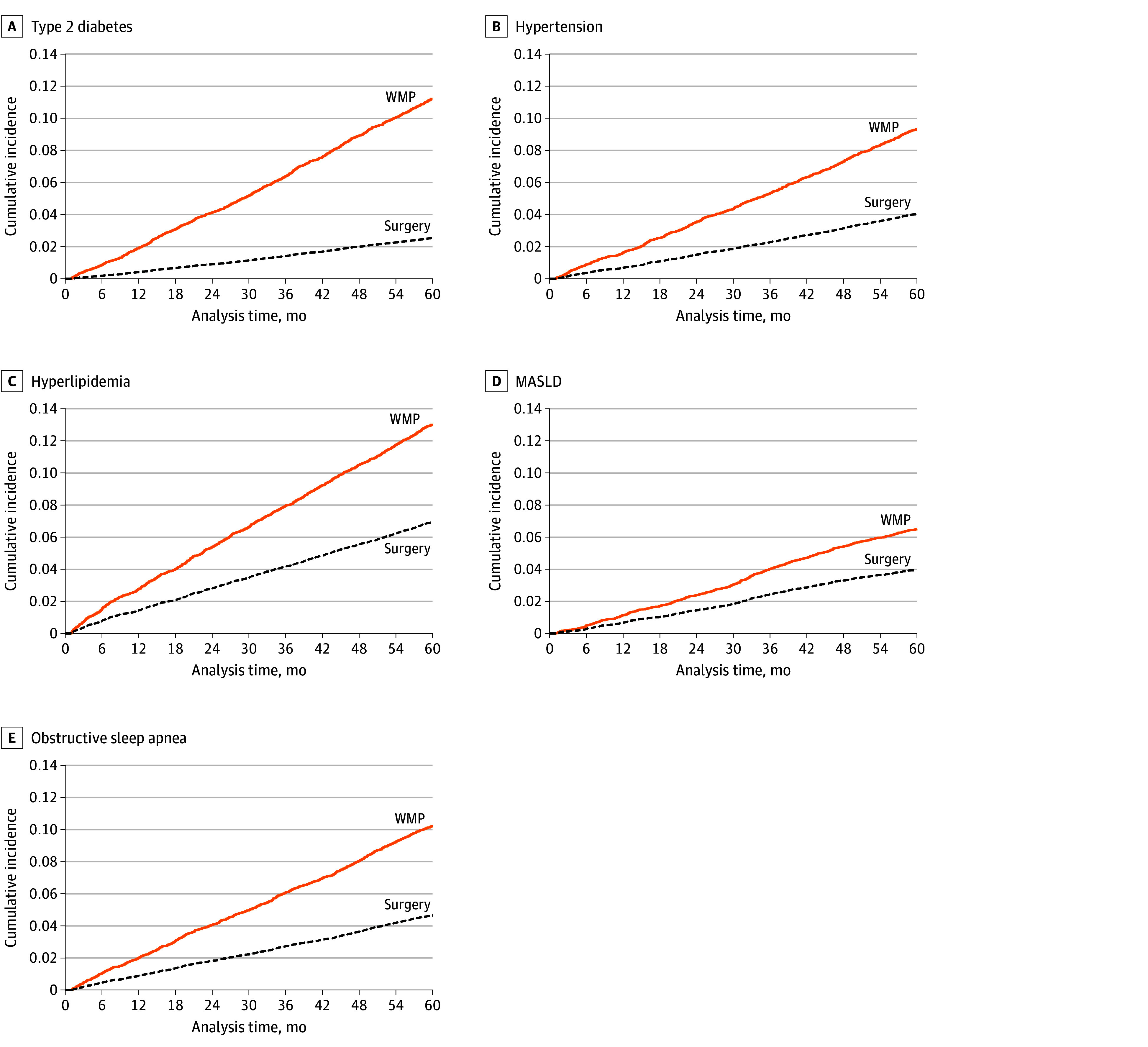
Cumulative Incidence Function Plot for Each Outcome Adjusted incidences of each comorbidity over a 5-year period. MASLD indicates metabolic dysfunction–associated steatotic liver disease; WMP, weight management program.

In adjusted Cox proportional hazards regression models after PSM, bariatric surgery was consistently associated with a decreased hazard of comorbidity development vs the WMP (Table 2). Patients in the bariatric surgery group were 79.2% less likely to develop T2D (HR, 0.21; 95% CI, 0.18-0.26; *P* < .001), 58.8% less likely to develop hypertension (HR, 0.41; 95% CI, 0.33-0.51; *P* < .001), 50.5% less likely to develop hyperlipidemia (HR, 0.49; 95% CI, 0.42-0.58; *P* < .001), 40.4% less likely to develop MASLD (HR, 0.60; 95% CI, 0.49-0.73; *P* < .001), and 56.9% less likely to develop OSA (HR, 0.43; 95% CI, 0.35-0.52; *P* < .001) (Figure 2; eFigure 5 in Supplement 1).

### Subgroup Analysis

When limiting the subcohorts to female veterans, those who underwent bariatric surgery vs enrolled in the WMP demonstrated a significantly decreased hazard of comorbidity development (eg, T2D: HR, 0.26; 95% CI, 0.17-0.39; *P* < .001) (eTable 3 in Supplement 1). The findings were similar to those in the primary analysis.

## Discussion

In this large retrospective study, veterans who underwent bariatric surgery had significantly decreased risk of developing major metabolic comorbidities compared with well-matched veterans enrolled in the WMP, with the greatest risk reductions observed in patients with T2D and hypertension. Results were consistent with findings from a subgroup analysis restricted to females.

Both randomized clinical trials and observational cohort studies have demonstrated the long-term benefits of bariatric surgery for cardiovascular disease risk factors, glycemic control, and blood pressure, as well as its role in reduced risk of mortality, myocardial infarction, heart failure, and stroke.^28,29,30,31,32^ Similarly, compared with medical or lifestyle interventions, bariatric surgery offers patients both a higher rate of comorbidity remission and higher likelihood of de-escalating daily medications.^33,34,35,36^ Mental health also improves after bariatric surgery, as studies have shown a greater health-related quality-of-life score in the years following surgery.^37^

However, there remains a paucity of literature on the consequences of forgoing or delaying bariatric surgery in terms of incident metabolic comorbidity development. A small, prospective study from Sweden in 1999 showed both short-term (1-2 years) and long-term (10-year) decreased incidence of diabetes, hypertension, and hypertriglyceridemia in patients who underwent bariatric surgery compared with patients who underwent conventional obesity treatment or medical weight loss.^38,39^ A recent study of nearly 71 000 bariatric surgery patients in the Swedish Patient Registrar matched to nonsurgical controls found an 85% decreased risk of developing diabetes among the surgery cohort.^40^ Our study corroborates these previous results and extends findings to additional metabolic comorbidities in a large North American cohort while accounting for confounding related to key covariates.

Our findings have important clinical implications. First, incident development of metabolic comorbidities is understudied in the bariatric surgery literature and represents a major outcome important to patients. The ability to provide prognostic counseling regarding future risk of metabolic comorbidities with and without bariatric surgery is crucial given the substantial health, psychological, and economic burdens associated with obesity-related comorbidities. Second, a better understanding of the risk of future metabolic comorbidities may support more informed decision-making about bariatric surgery. Third, these results highlight an additional important end point (incident metabolic comorbidities) that may be used in future prospective trials addressing weight loss given high observed baseline incidence rates. Fourth, these findings suggest that risk mitigation of metabolic comorbidities may contribute to the long-term advantages of bariatric surgery that have been demonstrated previously, including reduction in cardiovascular and oncologic risk,^32,41,42,43^ which strengthens the mechanistic understanding of the benefits of bariatric surgery. Fifth, this research lays the foundation for the development of models for estimating individual risk of specific comorbidities among patients eligible for bariatric surgery. Such models are critical for enhancing the decision-making by patients and clinicians alike, allowing for more tailored and informed treatment planning.

### Limitations

This study has several important limitations. Misclassification of exposures and outcomes is possible, although we used validated algorithms and fee basis tables to minimize misclassification. However, some surgeries performed outside of the VHA may not have been captured, potentially biasing the results toward the null. Despite careful PSM, residual confounding may persist. Insurance requirements mandate several months of enrollment in a medical weight loss program for bariatric surgery eligibility, and although many VHA patients were enrolled in the WMP prior to surgery, some may have participated in both the WMP and other weight loss programs. Additionally, prior research has shown highly variable engagement with the WMP itself, which may not be fully captured in our analysis.^13^ We also did not account for confounding by antiobesity medications, an important consideration given recent advances in pharmacotherapy. We did not stratify or categorize BMI in our primary analyses, which may overlook effect modification by baseline BMI and warrants further investigation. Finally, the VHA cohort was older and more male predominant than typical bariatric surgery cohorts, which may limit external validity. However, a subgroup analysis involving only female veterans showed similar findings. Because 37.1% of patients who underwent bariatric surgery were not eligible for PSM, generalizability to patients with greater baseline comorbidity may be limited, although similar associations may be expected in this group. The veteran population remains understudied in bariatric surgery research, highlighting the importance of our investigation.

## Conclusions

In this cohort study, patients who underwent bariatric surgery showed a significantly decreased risk of developing major metabolic comorbidities compared with patients who were enrolled in the medical WMP. These findings not only support the continued use of bariatric surgery as a durable, long-term solution for weight loss but also highlight its importance as a sustainable risk-mitigation strategy for individuals with obesity.

## Notes

Supplement 1. **eTable 1.** Bariatric Surgery Procedure Codes **eTable 2.** Validated Algorithms for Each Outcome **eTable 3.** Adjusted Hazard Ratios for Subgroup Analysis **eFigure 1.** Consort Diagram **eFigure 2.** Example of Risk Set Matching **eFigure 3.** Covariate Balance Achieved Through Propensity Score Matching for Each Obesity Related Comorbidity **eFigure 4.** Unadjusted Incidences of Each Comorbidity Represented by Heat Plot **eFigure 5.** Adjusted Incidences of Each Comorbidity Represented by Heat Plot
eReferences

Supplement 2.
Data Sharing Statement
