# Supplementary material for: Bariatric Surgery and Incident Development of Obesity-Related Comorbidities
Source: JAMA Netw Open. 2025 Sep 9;8(9):e2530787. doi: 10.1001/jamanetworkopen.2025.30787 (PMC12421336; doi:10.1001/jamanetworkopen.2025.30787)
Supplement: Supplement 1. — eTable 1. Bariatric Surgery Procedure Codes eTable 2. Validated Algorithms for Each Outcome eTable 3. Adjusted Hazard Ratios for Subgroup Analysis eFigure 1. Consort Diagram eFigure 2. Example of Risk Set Matching eFigure 3. Covariate Balance Achieved Through Propensity Score Matching for Each Obesity Related Comorbidity eFigure 4. Unadjusted Incidences of Each Comorbidity Represented by Heat Plot eFigure 5. Adjusted Incidences of Each Comorbidity Represented by Heat Plot eReferences [file jamanetwopen-e2530787-s001.pdf]

Supplemental Online Content

Bader AL, Hsu JY, Altieri MS, et al. Bariatric surgery and incident development of obesity-related comorbidities. *JAMA Netw Open*. 2025;8(9):e2530787. doi:10.1001/jamanetworkopen.2025.30787

eTable 1. Bariatric Surgery Procedure Codes

eTable 2. Validated Algorithms for Each Outcome

eTable 3. Adjusted Hazard Ratios for Subgroup Analysis

eFigure 1. Consort Diagram

eFigure 2. Example of Risk Set Matching

eFigure 3. Covariate Balance Achieved Through Propensity Score Matching for Each Obesity Related Comorbidity

eFigure 4. Unadjusted Incidences of Each Comorbidity Represented by Heat Plot

eFigure 5. Adjusted Incidences of Each Comorbidity Represented by Heat Plot

eReferences

This supplemental material has been provided by the authors to give readers additional information about their work.

© 2025 Bader AL et al. *JAMA Network Open*

eTable 1: Bariatric surgery procedure codes

Procedure	***CPT code*** (1)
Roux-en-Y gastric bypass (open, laparoscopic)	43644, 43645, 43846, 43847
Sleeve gastrectomy	43775

© 2025 Bader AL et al. *JAMA Network Open*

eTable 2: Validated algorithms for each outcome

Exposure / Outcome	ICD9/ICD10 codes	Algorithm	Reference
HTN	ICD9: 401.0, 401.1, 401.9, 402.xx, 403.xx, 404.xx<br>ICD10: I10.x, I11.x, I12.x, I13.x	2 or more separate visits/encounters with ICD 9/10 code (no specific time window)	**Yamada et al** (2) – used this in VA population<br>**Bhandari et al** (3) – used in non-VA population, validated in Kaiser
HLD	ICD9: 272.0, 272.1, 272.2, 273.4, 272.4<br>ICD10: E78.0, 78.1, 78.2, 78.3, 78.4, 78.5, 78.6	2 or more separate visits/encounters with ICD 9/10 code over 2 years	**Borzecki et al** (4) – VA validated
DM	ICD9: 250.x1, 250.x3 (T1DM), 250.0 – 250.9, 250.x0, 250.x1, 250.x2, 250.x3 (T2DM)<br>ICD10: E10.x (T1DM), E11.x (T2DM)	2 or more separate visits/encounters with ICD 9/10 code over 2 years	**Borzecki et al** (4) – VA validated<br>**Miller et al** (5) – VA validated
OSA	ICD9: 327.2, 327.21, 327.22, 327.23, 327.24, 327.25, 327.26, 327.27, 327.29, 780.51, 780.53. 780.57, 786.04<br>ICD10: G47.3, G47.30, G47.31, G47.38, E66.2, R06.3	2 or more separate visits/encounters with ICD 9/10 code (no specific time window)	**Keenan et al** (6) – non-VA validated
MASLD		ALT>40 for men or ALT>30 for women on two occasions at least 6 months apart, within 2 years of one another	**Serper et al** (7) – VA validated

© 2025 Bader AL et al. *JAMA Network Open*

eTable 3: Adjusted Hazard Ratios for Subgroup Analysis

Comorbidity	Adjusted HR	p-value
Diabetes	0.26 (0.17 – 0.39)	<0.001
Hypertension	0.47 (0.34 – 0.67)	<0.001
Hyperlipidemia	0.36 (0.26 – 0.50)	<0.001
Obstructive sleep apnea	0.35 (0.25 – 0.49)	<0.001
Metabolic dysfunction associated steatotic liver disease	0.69 (0.49 – 0.96)	0.029

© 2025 Bader AL et al. *JAMA Network Open*

eFigure1: Consort Diagram

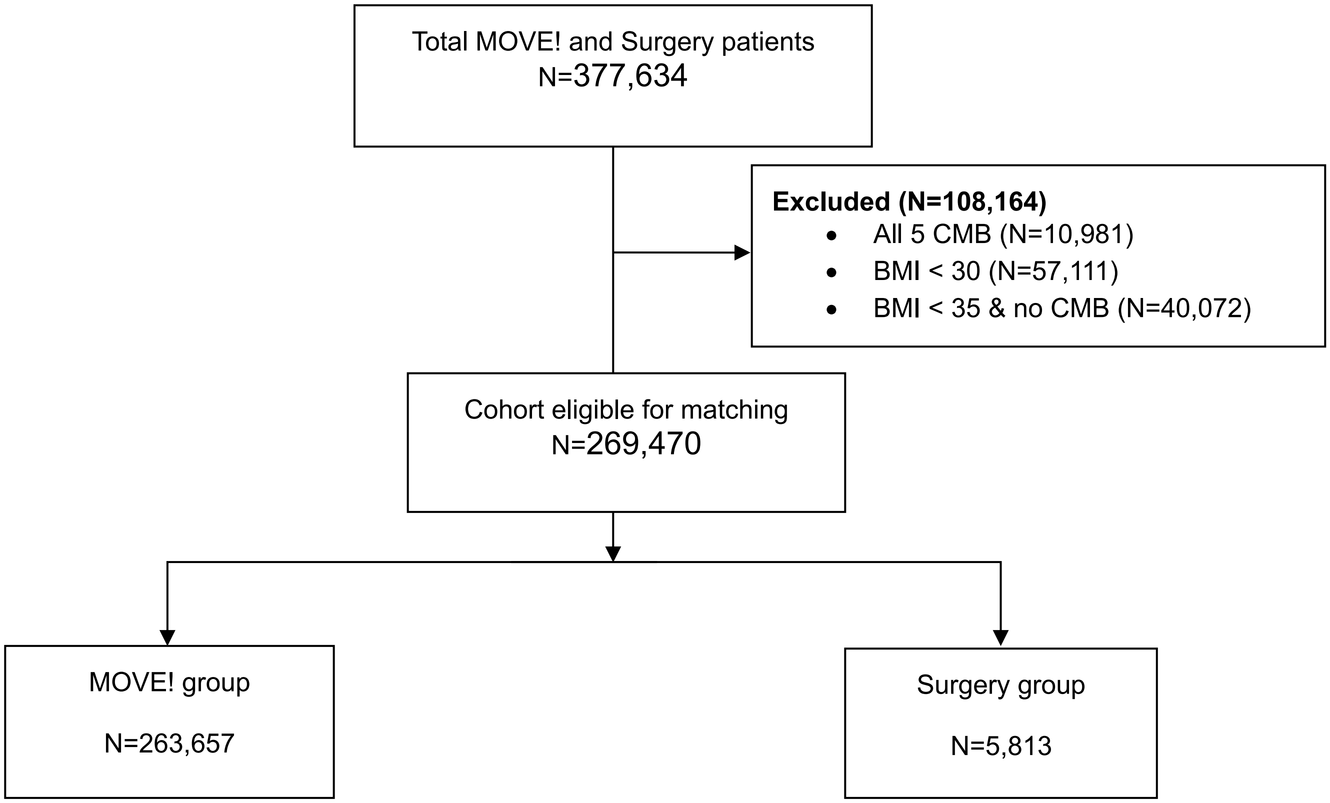

© 2025 Bader AL et al. *JAMA Network Open*

eFigure 2: Example of risk set matching

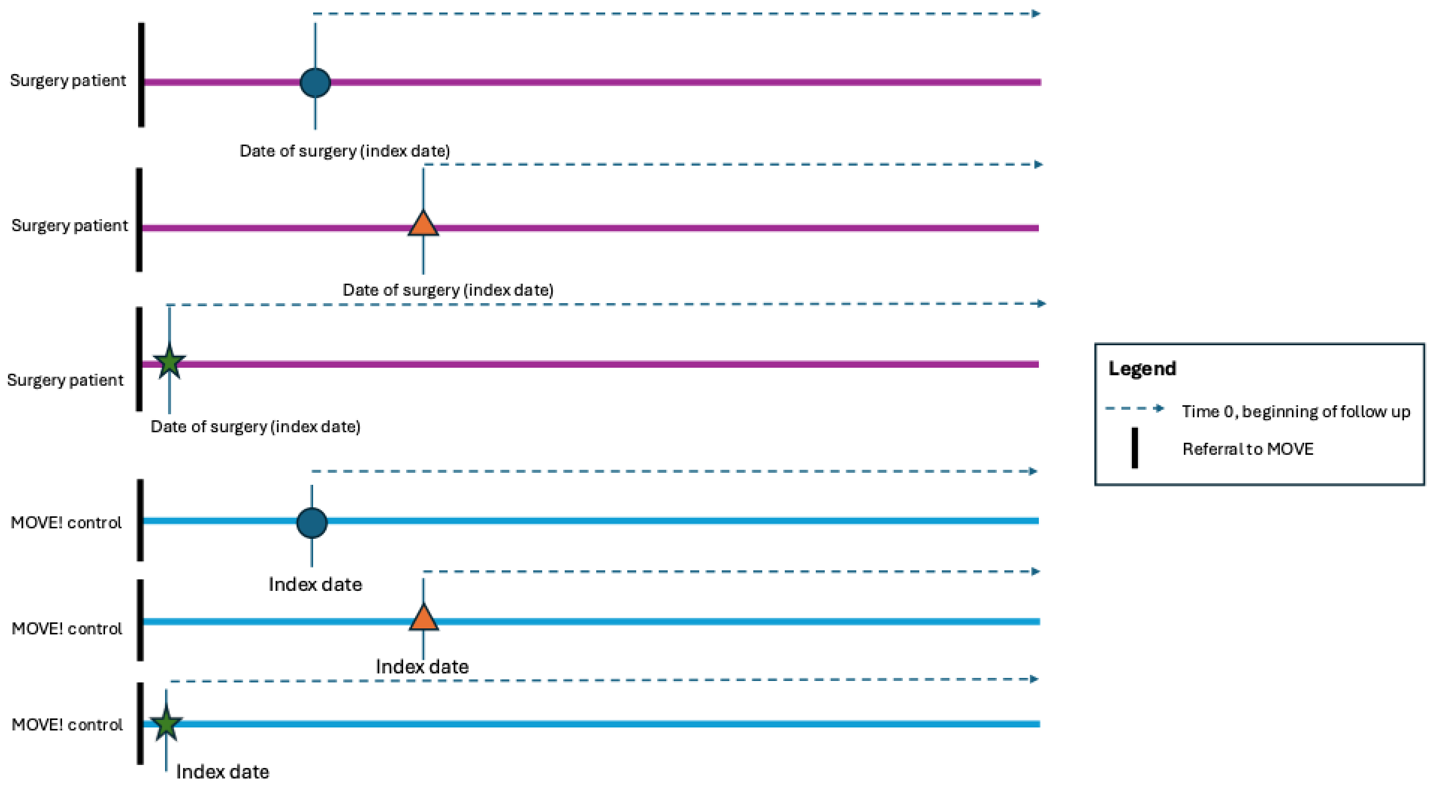

Figure depicts examples of the risk-set matching. Surgery patients were only matched to MOVE! patients who spent the equivalent amount of time in MOVE! or longer. Each symbol (circle, triangle, star) represents a different matched set. The dotted arrow represents beginning of follow up time (time 0) and the black line represents date of referral to MOVE! The index date for the surgery patients was date of surgery and the index date for MOVE! controls was date of enrollment.

© 2025 Bader AL et al. *JAMA Network Open*

eFigure 3: Covariate Balance Achieved through Propensity Score Matching for Each Obesity Related Comorbidity

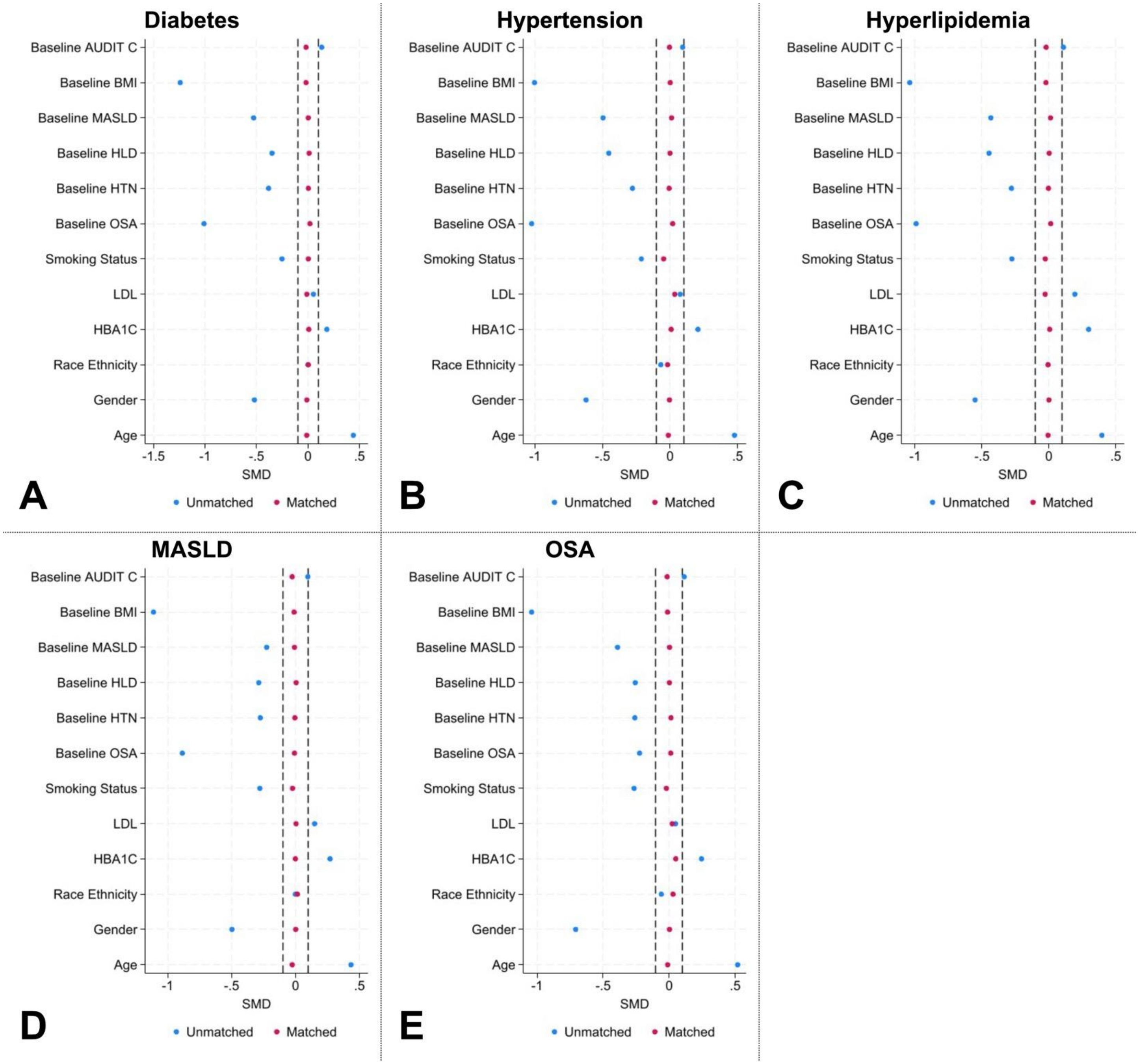

Pre-matching covariate balance represented by the blue dot; post-matching covariate balance is represented by the red dot. The dotted line corresponds to a standardized mean difference (SMD) between -0.1 and 0.1, which represents excellent covariate balance. Panel A) covariate balance for outcome of diabetes B) covariate balance for outcome of hypertension C) covariate balance for outcome of hyperlipidemia D) covariate balance for outcome of metabolic dysfunction associated steatotic liver disease (MASLD), E) covariate balance for outcome of obstructive sleep apnea (OSA)

© 2025 Bader AL et al. *JAMA Network Open*

eFigure 4: Unadjusted Incidences of Each Comorbidity Represented by Heat Plot

Unadjusted incidences of each comorbidity over a 5-year time period represented by heat plot. The darker colors represent higher incidences.

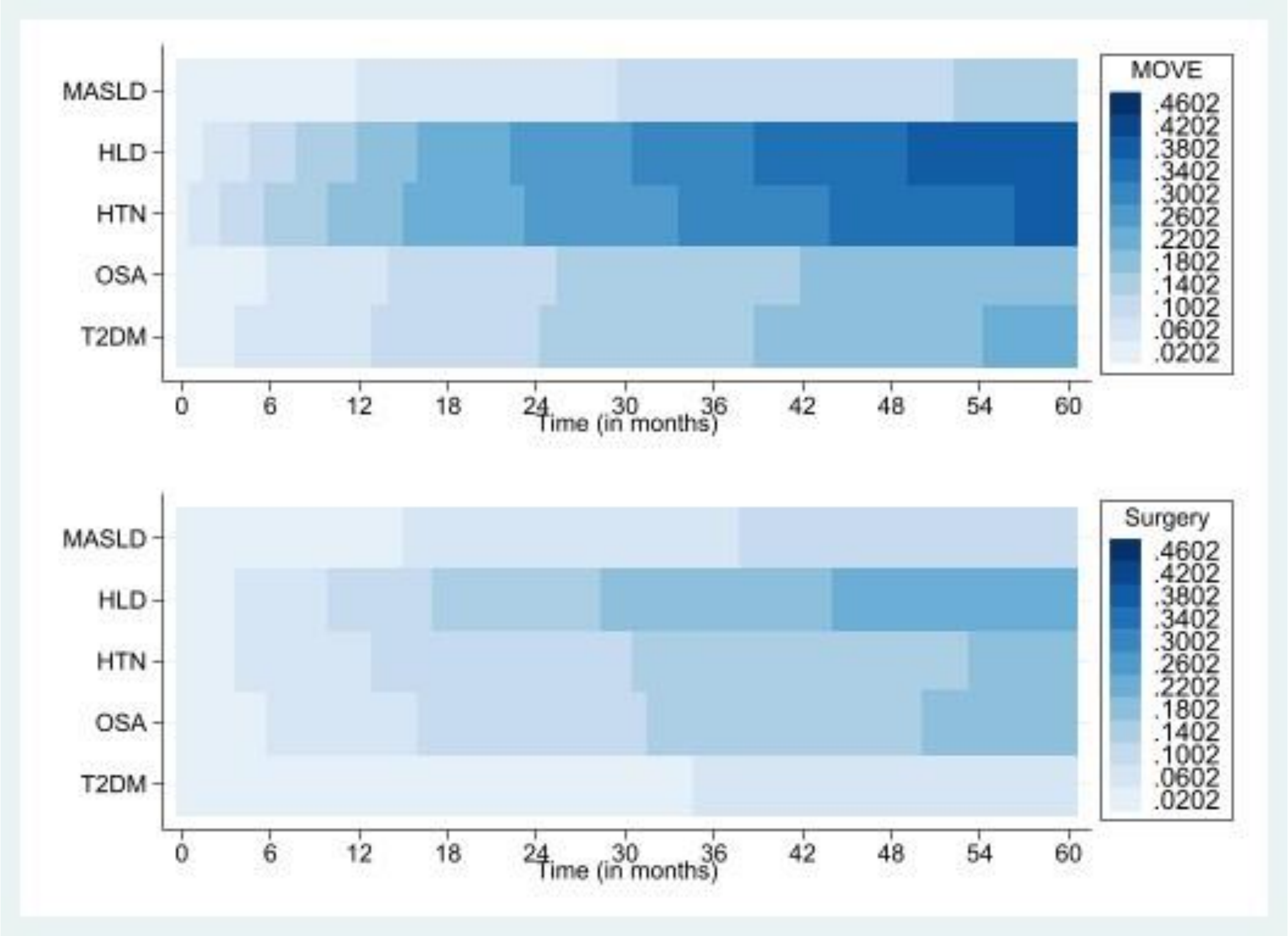

© 2025 Bader AL et al. *JAMA Network Open*

eFigure 5: Adjusted Incidences of Each Comorbidity Represented by Heat Plot

Adjusted incidences of each comorbidity over a 5-year time period represented by heat plot. The darker colors represent higher incidences.

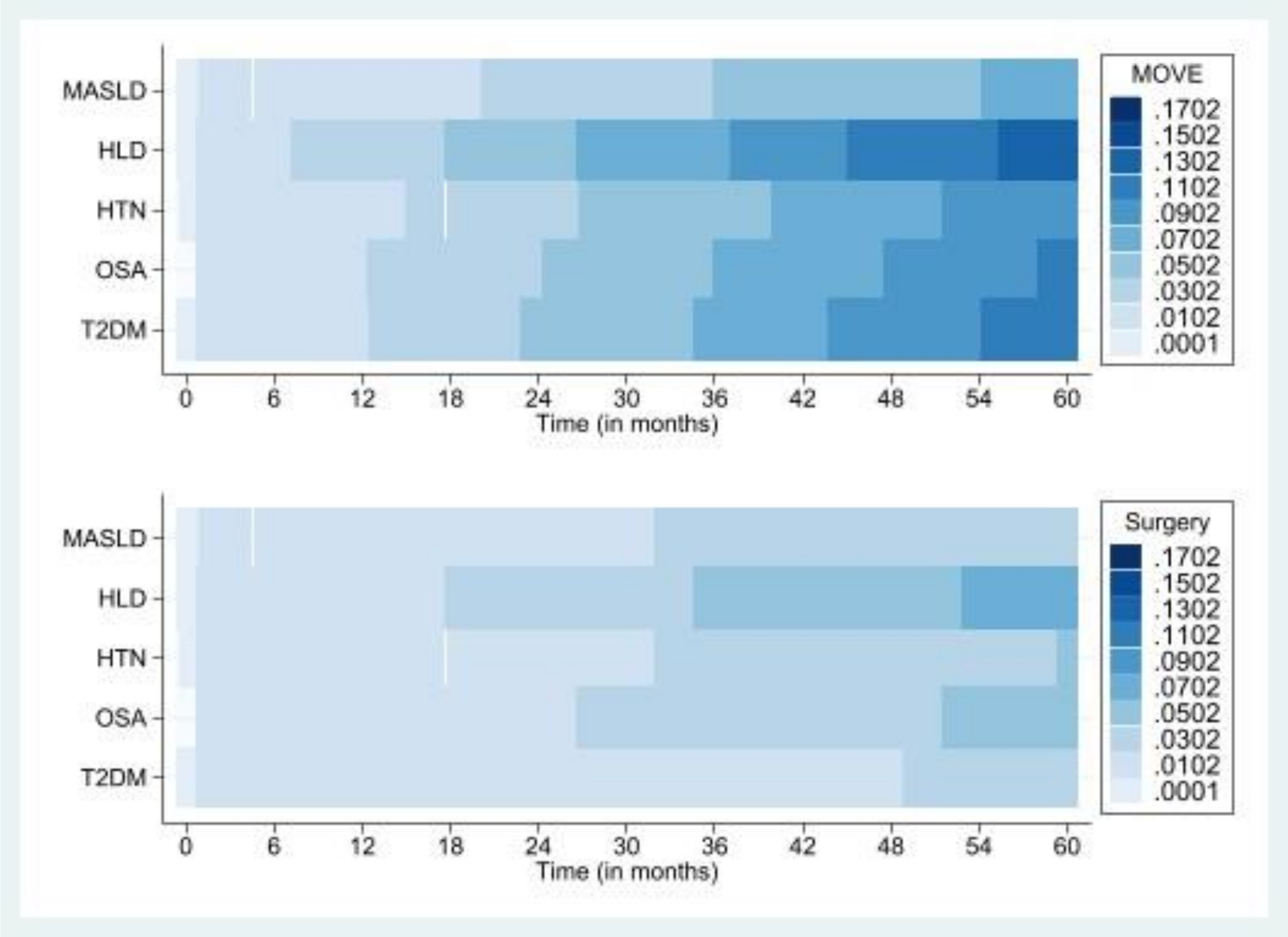

© 2025 Bader AL et al. *JAMA Network Open*

eReferences

1. Association AM. CPT Professional Codebook [Available from: https://www.cms.gov/medicare/regulations-guidance/physician-self-referral/list-cpt-hcpcs-codes.
2. Yamada M, Wachsmuth J, Sambharia M, Griffin BR, Swee ML, Reisinger HS, et al. The prevalence and treatment of hypertension in Veterans Health Administration, assessing the impact of the updated clinical guidelines. Journal of Hypertension. 9900:10.1097/HJH.0000000000003424.
3. Bhandari SK, Pashayan S, Liu ILA, Rasgon SA, Kujubu DA, Tom TY, et al. 25-Hydroxyvitamin D Levels and Hypertension Rates. The Journal of Clinical Hypertension. 2011;13(3):170-7.
4. Borzecki AM, Wong AT, Hickey EC, Ash AS, Berlowitz DR. Identifying hypertension-related comorbidities from administrative data: what's the optimal approach? Am J Med Qual. 2004;19(5):201-6.
5. Miller DR, Safford MM, Pogach LM. Who has diabetes? Best estimates of diabetes prevalence in the Department of Veterans Affairs based on computerized patient data. Diabetes Care. 2004;27 Suppl 2:B10-21.
6. Keenan BT, Kirchner HL, Veatch OJ, Borthwick KM, Davenport VA, Feemster JC, et al. Multisite validation of a simple electronic health record algorithm for identifying diagnosed obstructive sleep apnea. Journal of Clinical Sleep Medicine. 2020;16(2):175-83.
7. Serper M, Vujkovic M, Kaplan DE, Carr RM, Lee KM, Shao Q, et al. Validating a non-invasive, ALT-based non-alcoholic fatty liver phenotype in the million veteran program. PLOS ONE. 2020;15(8):e0237430.

© 2025 Bader AL et al. *JAMA Network Open*
