# Supplementary material for: Bariatric Surgery and Incident Development of Obesity-Related Comorbidities
Source: JAMA Netw Open. 2025 Sep 9;8(9):e2530787. doi: 10.1001/jamanetworkopen.2025.30787 (PMC12421336; doi:10.1001/jamanetworkopen.2025.30787)
Supplement: Supplement 2. — Data Sharing Statement [file jamanetwopen-e2530787-s002.pdf]

Data Sharing Statement

Bader. Bariatric Surgery and Incident Development of Obesity-Related Comorbidities. *JAMA Netw Open*. Published September 09, 2025. doi:10.1001/jamanetworkopen.2025.30787

Data
Data available: No

Additional Information
Explanation for why data not available: The data were extracted from the VHA CDW using structured query language (SQL), and data cleaning and analysis were performed using SQL and Stata. Due to privacy and security restrictions, sharing of raw VHA data is not permitted; however, the scripts for cleaning and analysis can be made available upon reasonable request, in accordance with VA data governance policies and privacy protections, to ensure transparency and reproducibility.
